# Late gadolinium enhancement by cardiac magnetic resonance imaging predicts reverse remodeling in patients with non-ischemic cardiomyopathy receiving β blocker therapy

**DOI:** 10.1186/1532-429X-13-S1-P294

**Published:** 2011-02-02

**Authors:** Keisuke Kida, Yasuyuki Kobayashi

**Affiliations:** 1 St. Marianna University School of Medicine, Kawasaki, Japan

## Introduction

β blockers improve left ventricular function and reduce morbidity and mortality in patients with chronic systolic heart failure. Not all patients, however, respond uniformly to this therapy. Cardiac magnetic resonance (CMR) imaging with late gadolinium enhancement (LGE) can detect a small and focal myocardial abnormality.

## Purpose

The aim of this study was to determine the predict factors of the therapeutic response in left ventricular ejection fraction (LVEF) and remodeling in patients with non-ischemic cardiomyopathy who were receiving treatment with β blockers. We hypothesized LGE by CMR imaging predicted therapeutic response in LVEF and remodeling in patients with non-ischemic cardiomyopathy who were receiving treatment with β blockers.

## Methods

This study included 19 heart failure patients (mean age 52 years, 15 men) who had non-ischemic origin confirmed by coronary angiography and underwent LGE by CMR imaging at 1 month after the initiation of β blocker therapy. Of these, 16 patients received Carvedilol 10 mg/day and 3 patients received Metoprolol 4 mg/day at 1 month of the beta blocker therapy. All patients were treated with a standard heart failure regimen of angiotensin-converting enzyme (ACE) inhibitor or angiotensin receptor blocker (ARB). LVEF was measured according to the Simpson’s modified method using echocardiography at 1 and 6 months.

## Results

Of the 19 patients, 11 (58 %) patients had positive LGE images. Overall, LVEF significantly increased (27 ± 8 to 47 ± 11 %, p<0.0001) and BNP significantly decreased (275 ± 281 to 70 ± 92 pg/ml, p<0.0001). The improvement of LVEF significantly differed between the LGE positive (13 ± 8 %) and negative (31 ± 15 %) patients (p=0.004).

## Conclusions

LGE by CMR imaging predicted therapeutic response in LVEF and remodeling in patients with non-ischemic cardiomyopathy treated with β blockers.

